# 

**Published:** 1907-01-19

**Authors:** 


### BOOKS RECEIVED.

T. B. Browne, Ltd.

"The Advertiser's A.B.C.," 1907. A most useful book of reference for advertisers and journalists.

The Health Resorts Bureau.

"French Law and Customs for the Anglo-Saxon." By A. S. Browne.

John Murray.

"Licensing and Temperance in Sweden, Norway, and Denmark."

Kegan Paul, Trench and Co.

"The Mind and the Brain." By Alfred Binet.

J. Willing, jun., Ltd.

"Willing's Press Guide," 1907.

The Caxton Publishing Co.

"The Nursling." By Pierre Budin. Translated by W. J. Maloney, M.B.

Methuen and Co.

"The Control of a Scourge." By C. P. Childe, B.A.

THERAPEUTICAL SOCIETY'S MEETINGS.—Tuesday, January 22, 1907:—Therapeutical Society, in the Apothecaries' Hall, Blackfriars, E.C., at 4.30 P.M., the following papers will be read: Dr. R. B. Wild, "The Proper Scope of the Teaching of Materia Medica, Pharmacology, and Therapeutics in the Medical Curriculum"; Dr. Bonnefin, "On a New Method of Rendering Creosote, and Cannabis Indica, Soluble"; and "Short Notes on Certain Useful but Little-known Drugs."

### NOTICE TO CORRESPONDENTS.

All MSS., Letters, Books for Review, and other matters intended for the Editor should be addressed THE EDITOR, "THE HOSPITAL," THE HOSPITAL BUILDINGS, 28 & 29 SOUTHAMPTON STREET, STRAND, W.C.

The Editor cannot undertake to return rejected MS., even when accompanied by stamped directed envelope.

### Notice to Advertisers and Subscribers.

All Advertisements, Orders for Copies of THE HOSPITAL, and Business Communications should be addressed to **THE MANAGER (Not to the Editor),** THE HOSPITAL, 28 & 29 Southampton Street, Strand London, W.C.

The Cost of Subscription, post free, is as follows:—Per week, 3½d.; per quarter, 3s. 3d.; per half-year, 6s. 6d.; per annum, 13s. Foreign and Colonial:—Per annum, 19s. 6d., payable, in all cases, in advance.

### New Books for Nurses

**Ready Shortly.** **2s. 6d. net, 2s. 9d. post free.**

### SKIN DISEASES:

### THEIR NURSING AND PRACTICAL MANAGEMENT.

By G. NORMAN MEACHAN, M.D.

**2s. 6d. net.** **2s. 9d. post free.**

### A MANUAL FOR NURSES ON ABDOMINAL SURGERY.

By HAROLD BURROWS, M.B., F.R.C.S.

*CONTENTS.*

Symptoms in Abdominal Disease.
Peritonitis.
Hernia.
Affections of the Stomach.
Affections of the Small Intestine.
Intestinal Obstruction.
Intussusception.
Appendicitis.
Affections of the Liver and Gall-bladder.
Diseases of the Uterus and Appendages.
Preparation for Abdominal Section.
After-troubles and Complications of Abdominal Operations.
Feeding.

**2s. net; 2s. 3d. post free.**

**You have often been in need of a Book**

That would aid you in emergency. That would help you to recollect some point you had forgotten or about which you had a doubt.

That would prove of assistance in difficulty.

That would convey information clearly and concisely on Diseases; their Symptoms and Nursing Treatment.

### THE NURSE'S "ENQUIRE WITHIN"

is a Pocket Encyclopædia of valuable nursing information alphabetically arranged, and

**Fills a Want**

that has long been felt by Nurses everywhere. It is bound in limp cloth boards with rounded corners, and its size and weight will admit of its being carried conveniently in the apron pocket without injury thereto.

**3d.; post free, 4½d.**

### THE CASE BOOK FOR PRIVATE NURSING,

CONTAINING RULINGS FOR

**Day and Night Nurses' Reports.**

Comprising Time, Nourishment, Stimulants, Medicines, Urine, Bowels, Summary Remarks, &c. &c.

**This Case Book has been described by some of the highest Medical Authorities as "the most concise Nurses' Report Book" issued.**

**The Scientific Press, Ltd.,**

*Catalogue of Nursing Manuals sent post free on application.*

**Publishers of Nursing Text-ooks, Charts, and Case-Books of all Descriptions.**

**28 & 29 SOUTHAMPTON STREET, STRAND, LONDON, W.C.**

